# Supplementary material for: HIVprotI: an integrated web based platform for prediction and design of HIV proteins inhibitors
Source: J Cheminform. 2018 Mar 9;10:12. doi: 10.1186/s13321-018-0266-y (PMC5845081; doi:10.1186/s13321-018-0266-y)
Supplement: Supplementary file 2 — Additional file 2. Source code of HIVProtI web server. [file 13321_2018_266_MOESM2_ESM.zip › HIVprotI_Source-code/hivprotI/weka-3-6-6/doc/weka/core/pmml/DefineFunction.html]

DefineFunction


---


|  |  |  |
| --- | --- | --- |
| **Overview** \| **Package** \| **Class** \| **Tree** \| **Deprecated** \| **Index** \| **Help** \| **Weka's home** | |  |
| **PREV CLASS**   **NEXT CLASS** | **FRAMES**    **NO FRAMES**     **All Classes** |  |
| SUMMARY: NESTED \| FIELD \| CONSTR \| METHOD | DETAIL: FIELD \| CONSTR \| METHOD |  |


---


## weka.core.pmml Class DefineFunction

```
java.lang.Object
  weka.core.pmml.Function
      weka.core.pmml.DefineFunction
```

**All Implemented Interfaces:**: java.io.Serializable

---

``` public class DefineFunction extends Function ```

Class encapsulating DefineFunction (used in TransformationDictionary).

**Version:**
:   $Revision 1.0 $

**Author:**
:   Mark Hall (mhall{[at]}pentaho{[dot]}com

**See Also:**: Serialized Form

---

**Constructor Summary**

`DefineFunction(org.w3c.dom.Element container,
weka.core.pmml.TransformationDictionary transDict)`


| **Method Summary** | |
| --- | --- |
| `Attribute` | `getOutputDef()`             Get the structure of the result produced by this function. |
| `java.lang.String[]` | `getParameterNames()`             Returns an array of the names of the parameters expected as input by this function. |
| `double` | `getResult(double[] incoming)`             Get the result of applying this function. |
| `void` | `setParameterDefs(java.util.ArrayList<Attribute> paramDefs)`             Set the structure of the parameters that are expected as input by this function. |
| `java.lang.String` | `toString()` |
| `java.lang.String` | `toString(java.lang.String pad)` |

**Methods inherited from class weka.core.pmml.Function**

`getFunction, getFunction, getName`

**Methods inherited from class java.lang.Object**

`equals, getClass, hashCode, notify, notifyAll, wait, wait, wait`

**Constructor Detail**

### DefineFunction

```
public DefineFunction(org.w3c.dom.Element container,
                      weka.core.pmml.TransformationDictionary transDict)
               throws java.lang.Exception
```

**Throws:**: `java.lang.Exception`


**Method Detail**

### getOutputDef

```
public Attribute getOutputDef()
```

:   Get the structure of the result produced by this function.

    :   **Specified by:**: `getOutputDef` in class `Function`
    :   **Returns:**: the structure of the result produced by this function.

---


### getParameterNames

```
public java.lang.String[] getParameterNames()
```

:   Returns an array of the names of the parameters expected
    as input by this function. May return null if this function
    can take an unbounded number of parameters (i.e. min, max, etc.).

    :   **Specified by:**: `getParameterNames` in class `Function`
    :   **Returns:**: an array of the parameter names or null if there are an unbounded number of parameters.

---


### getResult

```
public double getResult(double[] incoming)
                 throws java.lang.Exception
```

:   Get the result of applying this function.

    :   **Specified by:**: `getResult` in class `Function`
    :   **Parameters:**: `incoming` - the arguments to this function (supplied in order to match that of the parameter definitions **Returns:**: the result of applying this function. When the optype is categorical or ordinal, an index into the values of the output definition is returned. **Throws:**: `java.lang.Exception` - if there is a problem computing the result of this function

---


### setParameterDefs

```
public void setParameterDefs(java.util.ArrayList<Attribute> paramDefs)
                      throws java.lang.Exception
```

:   Set the structure of the parameters that are expected as input by
    this function. This must be called before getOutputDef() is called.

    :   **Specified by:**: `setParameterDefs` in class `Function`
    :   **Parameters:**: `paramDefs` - the structure of the input parameters **Throws:**: `java.lang.Exception` - if the number or types of parameters are not acceptable by this function

---


### toString

```
public java.lang.String toString()
```

:   **Overrides:**: `toString` in class `Function`

---


### toString

```
public java.lang.String toString(java.lang.String pad)
```

:   **Overrides:**: `toString` in class `Function`


---


|  |  |  |
| --- | --- | --- |
| **Overview** \| **Package** \| **Class** \| **Tree** \| **Deprecated** \| **Index** \| **Help** \| **Weka's home** | |  |
| **PREV CLASS**   **NEXT CLASS** | **FRAMES**    **NO FRAMES**     **All Classes** |  |
| SUMMARY: NESTED \| FIELD \| CONSTR \| METHOD | DETAIL: FIELD \| CONSTR \| METHOD |  |


---
